# Synergistic anti-inflammatory effect: simvastatin and pioglitazone reduce inflammatory markers of plasma and epicardial adipose tissue of coronary patients with metabolic syndrome

**DOI:** 10.1186/1758-5996-6-47

**Published:** 2014-03-31

**Authors:** Adriana Ferreira Grosso, Sérgio Ferreira de Oliveira, Maria de Lourdes Higuchi, Desidério Favarato, Luís Alberto de Oliveira Dallan, Protásio Lemos da Luz

**Affiliations:** 1 Heart Institute (InCor) HCFMUSP, University of São Paulo Medical School, São Paulo, Brazil; 2 Instituto do Coração do Hospital das Clínicas da Faculdade de Medicina da, Universidade de São Paulo, Av. Dr. Enéas de Carvalho Aguiar, 44, 5º andar, bloco II, sala 8 Cerqueira César, 05403-000 São Paulo, SP, Brazil

**Keywords:** Atherosclerosis, Epicardial adipose tissue, Inflammation, Pioglitazone, Simvastatin

## Abstract

**Background:**

The inappropriate secretion of adipocytokines plays a critical role in chronic inflammatory states associated with obesity-linked type 2 diabetes and atherosclerosis. The pleiotropic actions of simvastatin and pioglitazone on epicardial adipose tissue (EAT) are unknown. This study assessed the anti-inflammatory actions of simvastatin and pioglitazone on EAT in patients with coronary artery disease (CAD) and metabolic syndrome (MS).

**Methods:**

A total of 73 patients with multivessel CAD who underwent elective bypass grafting were non-randomly allocated to one of four subgroups: Control (n = 17), simvastatin (20 mg/day, n = 20), pioglitazone (15 mg or 30 mg/day, n = 18), or simvastatin + pioglitazone (20 mg/day + 30 mg/day, respectively, n = 18); 20 valvar patients were also included. EAT samples were obtained during surgery. The infiltration of macrophages and lymphocytes and cytokines secretion were investigated using immunohistochemical staining and compared to plasma inflammatory biomarkers.

**Results:**

Simvastatin significantly reduced plasma interleukin-6, leptin, resistin and monocyte chemoattractant protein-1 (p < 0.001 for all); pioglitazone reduced interleukin-6, tumoral necrose factor-alpha, resistin and matrix metalloproteinase-9 (p < 0.001 for all). Simvastatin + pioglitazone treatment further reduced plasmatic variables, including interleukin-6, tumoral necrose factor-alpha, resistin, asymmetric dimethylarginine and metalloproteinase-9 vs. the control group (p < 0.001). Higher plasma adiponectin and lower high sensitivity C-reactive protein concentrations were found simultaneously in the combined treatment group. A positive correlation between the mean percentage systemic and tissue cytokines was observed after treatments. T- and B-lymphocytes and macrophages clusters were observed in the fat fragments of patients treated with simvastatin for the first time.

**Conclusions:**

Pioglitazone, simvastatin or combination treatment substantially reduced EAT and plasma inflammatory markers in CAD and MS patients. These tissue effects may contribute to the control of coronary atherosclerosis progression.

## Background

Adipose tissue stores and releases energy, and it also exerts important autocrine/paracrine and endocrine functions, especially through the secretion of bioactive cytokines known as adipokines. Therefore, visceral fat accumulation causes inflammatory cells infiltration and cytokine expression. Adipose tissue macrophages play a critical role in chronic inflammatory states [1] that are associated with obesity-linked diseases, such as diabetes and atherosclerosis [2-4].

Epicardial adipose tissue (EAT) has received considerable attention. EAT is a recognized source of inflammatory cytokines, which may contribute to the pathogenesis of coronary atherosclerotic lesions [5]. Inflammatory mediators from extravascular adipocytes promote coronary artery disease, which may explain the increased cardiovascular risk in patients with insulin resistance [6], especially in obese individuals [7]. Several human studies indicate that pericardial fat deposits are more metabolically active than subcutaneous adipose tissue [5,6]. For example, EAT from patients undergoing coronary bypass grafting contained significantly more interleukin-1β (IL-1β), interleukin-6 (IL-6), monocyte chemoattractant protein-1 (MCP-1) and tumoral necrose factor-α (TNF-α) mRNA and protein than subcutaneous adipose tissue [5]. Cytokine concentrations in epicardial fat correlated with an accumulation of inflammatory cells, such as T-lymphocytes, macrophages and mast cells, in the vicinity of EAT. Epicardial mRNA levels for CD45, a marker of macrophage infiltration, were significantly increased compared to abdominal fat from patients subjected to bypass grafting [6]. The infiltration of macrophages and CD-8-positive T cells in the EAT of patients subjected to bypass was greater than patients who underwent surgery for aortic or mitral valve replacement [8]. Finally, patients with advanced CHD exhibited lower epicardial adiponectin levels, which may contribute to the increased cardiovascular risk [9-11].

Obesity predisposes humans and animals to an accumulation of excess epicardial fat [6,12], and its mass, assessed using transthoracic echocardiography and magnetic resonance in healthy subjects, may be a novel indicator of cardiovascular risk [13,14]. These facts support the importance of analyses of therapeutic interventions to interfere with EAT [15]. Pioglitazone and simvastatin have pleiotropic effects and reduce pro-inflammatory markers and improve insulin sensitivity [16-18]. However, no documentation of change in EAT inflammatory cell expression and adipokines after Simvastatin and/or Pioglitazone therapies in CAD patients with MS. This study assessed the anti-inflammatory effects of these compounds on EAT and correlated them with plasma/tissue inflammatory markers because tissue effect is an objective criteria for drug evaluations. Our findings demonstrated strong anti-inflammatory actions on EAT and excellent correlations between plasma and tissue effects.

## Methods

### Subjects

The Ethics Committee of the Heart Institute (InCor) and Hospital das Clinicas of the University of São Paulo Medical School approved the protocol. Seventy-three consecutive patients with multivessel CAD and MS who underwent bypass grafting and 20 valvar patients who underwent surgery for mitral valve replacement were enrolled. The attending heart team allocated the 73 CAD patients to one of four groups based on clinical and angiographic evaluation: control (n = 17), simvastatin alone (20 mg/day, n = 20), pioglitazone alone (30 mg/day, n = 18), or simvastatin + pioglitazone (20 mg/day + 30 mg/day, respectively, n = 18). Randomization was not necessary because the primary objective of the study was to correlate plasma and tissue effects rather than directly compare the two drugs. Treatments were initiated three months before surgery and maintained until the operation. All subjects who received pioglitazone were type 2 diabetics. No lipid-lowering drugs were administered in the control and pioglitazone groups, and statins were initiated during the in-hospital postoperative period. MS was defined according to NCEP-ATP III criteria [19,20]. Patients affected by liver disease, renal failure, neoplastic diseases, HIV positive, metabolic diseases, or those who smoked were excluded. Written informed consent was obtained from each patient.

#### Blood collection

Peripheral venous blood was drawn into pyrogen-free tubes with or without EDTA as an anticoagulant immediately before surgery after 10–12 h overnight fast. Plasma glucose was determined using spectrophotometric methods (Glucose Flex Reagent Cartridge, Dade Behring). An ELISA kit (R&D Systems) quantified adiponectin, leptin, resistin, MCP-1, matrix metalloproteinase-9 (MMP-9) and asymmetric dimethylarginine (ADMA) in human serum. IL-6, TNF-α and insulin concentrations in human serum were quantified using the enzyme immunometric assay, Immulite 2000. Quantification of serum HbA1c level was accomplished using the Hemoglobin A1c test Tinaquant II Roche/Hitachi system.

#### Immunohistochemical staining

Adipose tissue samples (average 0.5-1.0 g) were taken near the proximal tract of the right coronary artery at the beginning of surgery for cardiopulmonary bypass before heparin administration. Tissue was formalin-fixed, and paraffin-embedded tissue sections were deparaffinized in xylene and dehydrated in a graded series of ethanol. Endogenous peroxidase activity was quenched using 6% hydrogen peroxide, followed by incubation with CAS-Block™ Invitrogen for 10 min. Antigen retrieval was performed in Tris-ethylenediaminetetraacetic acid buffer (pH 9.0) for 10 min. Immunohistochemical staining of 5-μm-thick sections was performed using primary antibodies against CD20/lymphocytes B (1:1000, clone L26, Dako, Denmark), CD45/lymphocytes T (1:5, clone UCHL, Dako, Denmark), CD68/macrophages (1:320, clone KP-1, Dako, Denmark), alpha tumor necrosis factor (TNF-α) (1:20, clone 28401, R&D System, UK), interleukin-6 (IL-6) (1:10, clone 1936, R&D System, UK), adiponectin (1:2000, clone 6D, US Biological), leptin (1:10, clone AF 398, R&D System, UK) and resistin (1:50, clone LS-B2879, Lifespan, USA) followed by incubation with the secondary antibody Picture ™MAX Polymer Invitrogen. Localization of the primary antibody was visualized with 3.3′-diaminobenzidine and counter-stained with hematoxylin. The percentage of positive area for cells and inflammatory markers was counted in three fields (edges, center and adjacent to vessels) using an image analyzer (Leica Cambridge Quantimet) with a 20X objective in a double-blind fashion.

#### Statistical analysis

The data are expressed as the means ± standard deviation (SD). One-way ANOVA followed by Bonferroni test compared the mean values of continuous variables between treatment groups followed by post hoc analysis. The sample size provided a power of 0.80 at the alpha = 0.05 level of significance to detect a difference of 0.5 (expressed as adiponectin/actin ratio) in adiponectin protein expression between the CAD and valvar groups. Two-tailed p < 0.05 indicated statistical significance. Analyses were performed using SPSS version 19.

## Results

### Demographic data

Patient demographic characteristics are summarized in Table 1. CAD/MS patients were significantly older and had larger waist circumferences and higher initial and final weights than the valvar group. CAD/MS groups were homogeneous for males sex (65.7%) and waist circumference. All CAD/MS subjects were hypertensive, 10% suffered a myocardial infarction in the past, 2% underwent a coronary angioplasty and 2% had previous CABG. Left ventricular ejection fraction, as assessed using LV angiogram, was above 45% in all patients.

**Table 1:** Demographic characteristics

	**Valvar (n = 20)**	P* value	**CAD/MS group**				
			**Control (n = 17)**	**Simvastatin (n = 20)**	**Pioglitazone (n = 18)**	**Pioglitazone + Simvastatin (n = 18)**	P** value
**Age (yrs)**	52.3 ± 8.0	<0.001	64.3 ± 6.06	60.1 ± 6.38	57.6 ± 5.89	60.1 ± 7.08	0.024
**Male (%)**	6(30)	0.005	12(70.6)	16(80)	10 (55.6)	10 (55.6)	0.314
**Waist circumference (cm)**	78.4 ± 11.9	<0.001	103.6 ± 6.43	103.2 ± 8.62	103.7 ± 8.09	105.2 ± 9.06	0.883
**Systolic BP (mmHg)**	113 ± 10.3	<0.001	145 ± 17.1	134 ± 15.1	162 ± 20.3	145 ± 13.8	<0.001
**Diastolic BP (mmHg)**	72 ± 11.5	0.006	87 ± 11.8	78 ± 10.3	85 ± 12	73 ± 11.2	0.001
**Baseline Weight (Kg)**	58.9 ± 12.4	<0.001	85.8 ± 13.7	76.7 ± 10.8	80.6 ± 13.7	83.9 ± 10.2	0.122
**Final Weight (Kg)**	57.2 ± 11.9	<0.001	82.5 ± 12.9	74.6 ± 10.5	81.4 ± 14.6	83.3 ± 9.3	0.102

Values are means ± SD or n(%). CAD = coronary artery disease; BP = blood pressure. P* value = Comparison between non-CAD group and CAD/MS group. P** value = Comparison between CAD/MS groups.

### Laboratory data

Table 2 summarizes final laboratory values. The majority of CAD/MS groups had higher triglycerides, HDL-c, glucose, HbA1c, leptin, resistin and MCP-1 plasma levels than valvar patients. Valvar patients showed higher plasma adiponectin than patients with CAD/MS (p < 0.001).

**Table 2:** Laboratory measurements

	**Valvar (n = 20)**	P* value	**CAD/MS group**				
			**Control (n = 17)**	**Simvastatin (n = 20)**	**Pioglitazone (n = 18)**	**Pioglitazone + Simvastatin (n = 18)**	P** value
**TG (mg/dL)**	103.1 ± 40.48	0.002	162.88 ± 55.89	152 ± 68.64	167.89 ± 61.29	110.39 ± 22.1	0.011
**LDL-c (mg/dL)**	103.5 ± 29.68	0.381	123.76 ± 19.13	92.45 ± 33.57	142 ± 31.15	89.06 ± 25.62	<0.001
**HDL-c (mg/dL)**	44.1 ± 13.88	<0.001	34.35 ± 3.9	40.25 ± 7.5	41.39 ± 8.36	41.89 ± 6.33	0.006
**Glucose (mg/dL)**	82.95 ± 11.77	<0.001	101.18 ± 12.34	93.2 ± 17.11	120.44 ± 23.99*	144.44 ± 31.89*	<0.001
**HbA1c (%)**	5.63 ± 0.41	0.003	5.78 ± 0.49	5.59 ± 0.57	6.72 ± 1.55	6.41 ± 1.16	0.004
**Urea (mg/dL)**	17 ± 4.11	0.3	25 ± 7.5	21 ± 3.3	31 ± 2.22	30 ± 2.24	0.011
**Creatinine (mg/dL)**	0.9 ± 0.2	0.42	1.1 ± 0.8	1.0 ± 0.42	1.2 ± 0.9	1,25 ± 0.7	0.01
**hsCRP (mg/L)**	3.89 ± 1.49	0.466	7.42 ± 1.45	1.93 ± 1.59	2.97 ± 1.21	1.88 ± 0.65	<0.001
**IL-6 (pg/mL)**	3.51 ± 0.95	0.811	5.3 ± 1.06	3.29 ± 1.15	3.15 ± 1.15	2.68 ± 0.69	<0.001
**TNF-α (pg/mL)**	14.71 ± 4.06	0.317	16.58 ± 2.4	18.28 ± 10.91	7.92 ± 1.03	8.65 ± 2.1	<0.001
**Leptin (pg/mL)**	3519.75 ± 1997.54	<0.001	17043.69 ± 2065.78	4836.25 ± 1310.68	42899.54 ± 9011.58	13979.94 ± 1622.5	<0.001
**Resistin (ng/mL)**	4.34 ± 1.09	<0.001	12.1 ± 1.38	6.41 ± 0.97	5.41 ± 1.32	7.41 ± 0.99	<0.001
**Adiponectin (mg/mL)**	15280 ± 4389.45	<0.001	1480.59 ± 426.02	4601.85 ± 1044.07	6099.38 ± 1227.84	7427.43 ± 1229.81	<0.001
**MCP-1 (pg/mL)**	73 ± 6.23	<0.001	341.93 ± 66.16	66.48 ± 11.2	381.55 ± 69.28	325.98 ± 62.07	<0.001
**ADMA (μmol/L)**	0.47 ± 0.13	0.595	0,53 ± 0.14	0.47 ± 0.12	0.44 ± 0.33	0.34 ± 0.11	0.05
**MMP-9 (ng/mL)**	553.35 ± 157.38	0.146	577.08 ± 147.01	672.65 ± 340.37	216.54 ± 32.6	362.76 ± 103.57	<0.001

Values are means ± SD. TG = triglyceride; LDL = low-density lipoprotein cholesterol; HDL = high-density lipoprotein cholesterol; HbA1c = hemoglobin A1c. In the Pioglitazone only and Simvastatin + pioglitazone groups, the baseline values of glucose were higher than final values. hsCRP = C-reactive protein IL-6 = interleukin-6; TNF-α = tumoral necrosis factor. P* value = Comparison between non-CAD group and CAD/MS group. P** value = Comparison between CAD/MS groups.

### Treatment effects on blood parameters

Treatment with simvastatin alone, pioglitazone alone and simvastatin + pioglitazone significantly reduced plasma CRP in CAD/MS patients compared to the control group (p < 0.001). Simvastatin monotherapy significantly reduced plasma IL-6, leptin, resistin and MCP-1 (p < 0.001 for all), but pioglitazone monotherapy reduced IL-6, TNF-α, resistin and MMP-9 compared to the control group (p < 0.001 for all). Finally, simvastatin + pioglitazone treatment reduced IL-6, TNF-α, resistin, ADMA and MMP-9 compared to the control group (p < 0.001). All treatments increased adiponectin plasma levels (p < 0.001 for all). Higher plasma adiponectin and lower hsCRP concentrations were found simultaneously in the combined treatment group.

### Treatment effects on epicardial adipose tissue

**Figure 1:**
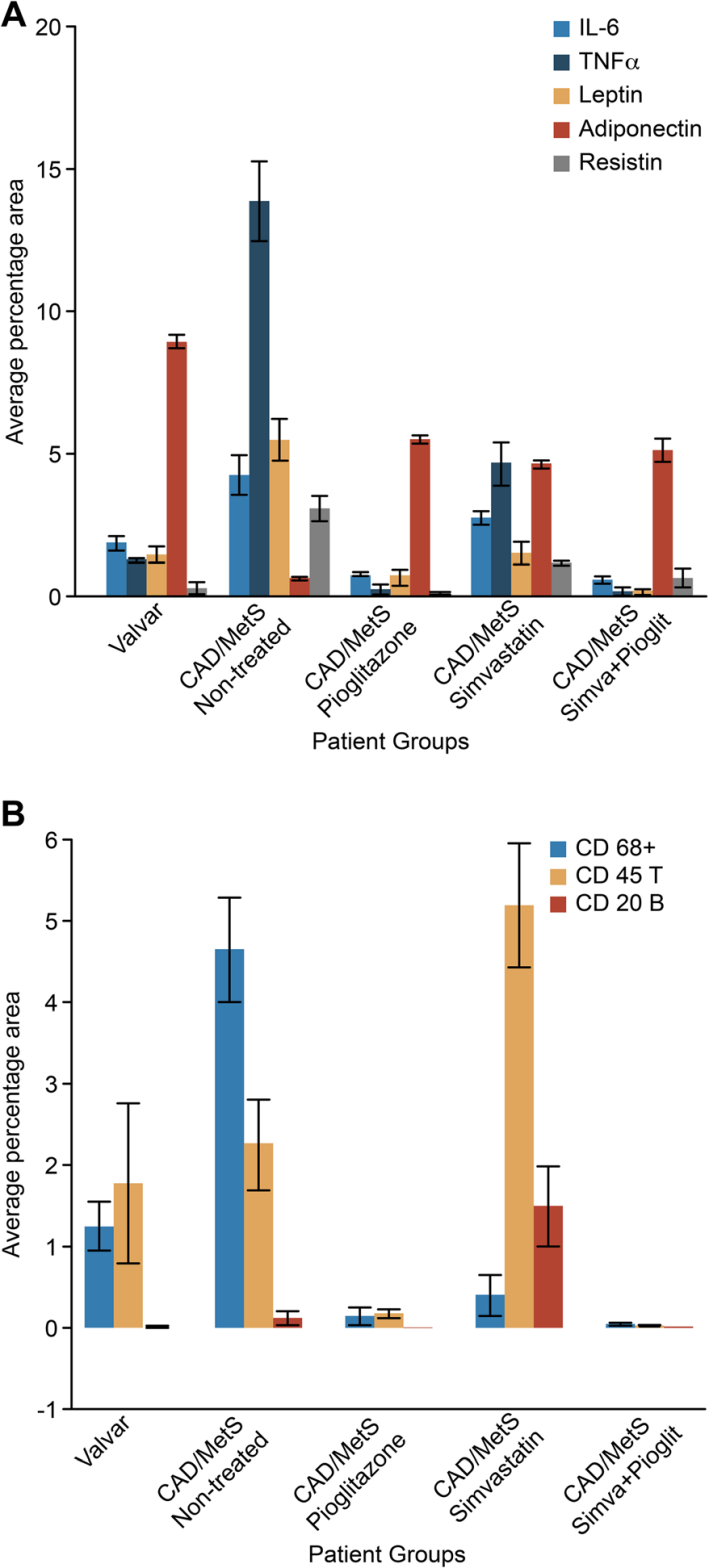
**Immunohistochemical staining in the EAT: quantify area. (A)** Average percentage area for pro- and anti-inflammatory cytokines in the EAT (P < 0.005). TNF-α: tumoral necrosis factor alpha; IL-6: interleukin 6. Analysis of variance one-way ANOVA; Bonferroni post-hoc test. **(B)** Average percentage area for CD68+ macrophages, CD45 T- and CD20 B-lymphocytes in EAT according to treatment group. (p < 0.05) for comparisons between CAD/MS groups, except for the comparison between the percentage of T-lymphocytes of pioglitazone and control groups (p = 0.236), and the percentage of macrophages using monotherapy with pioglitazone and simvastatin treatments (p = 0.349). CD45 T and CD20 B cells formed inflammatory clusters close to the edge of and around vessels of the EAT sections in the simvastatin group, but the center of the fat fragment was free of inflammatory cells. CD68+: macrophages; CD45T: T lymphocytes; CD20B: B lymphocytes. Analysis of variance: one-way ANOVA; Bonferroni post-hoc test. See text for details.

**Figure 2:**
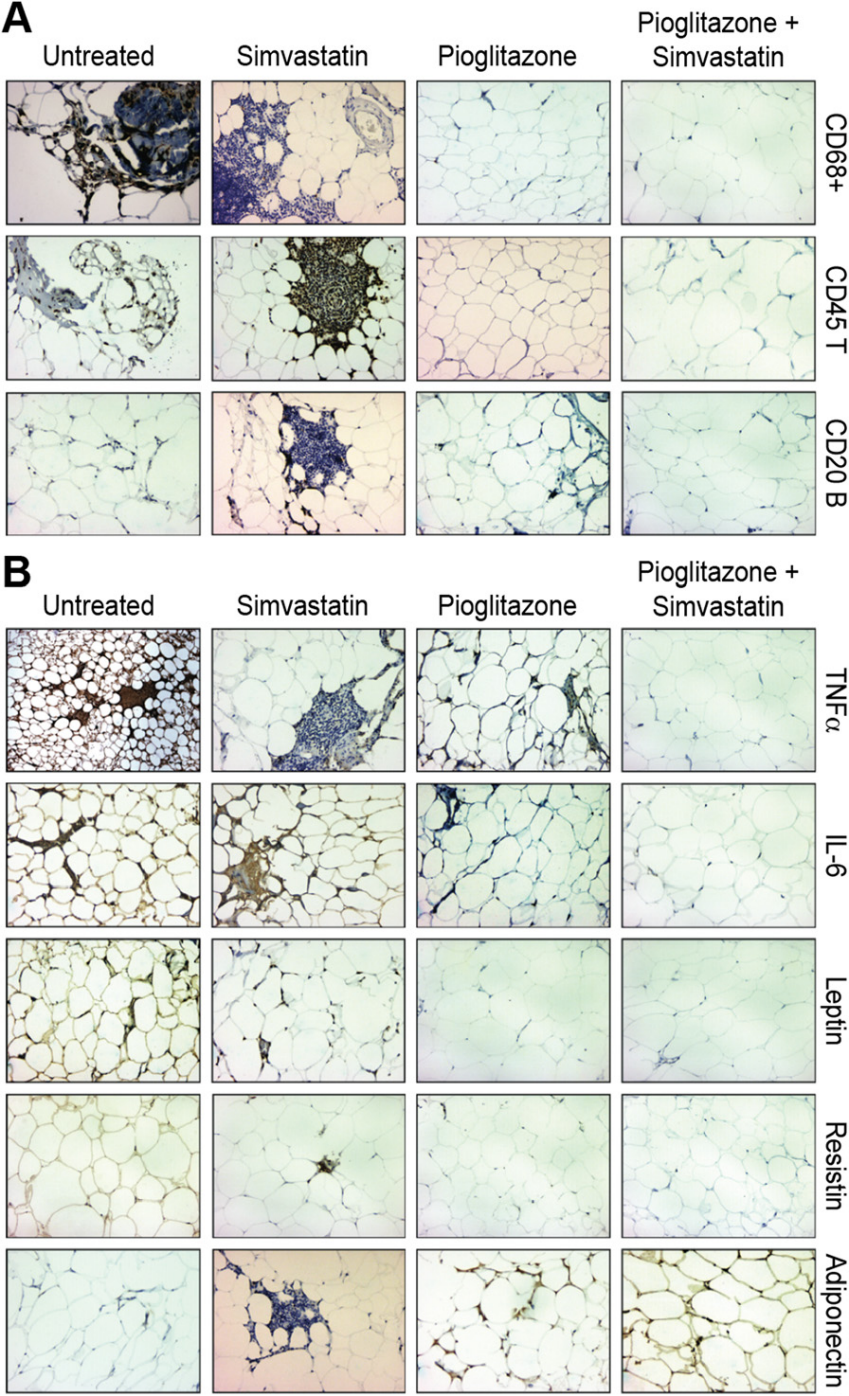
**Immunohistochemical staining in the EAT: components. (A)** Immunohistochemical staining for inflammatory cells in the EAT. CD68+: macrophages; CD45 T: T-lymphocytes; CD20 B: B-lymphocytes; TNF-α: tumoral necrosis factor alpha; IL-6: interleukin 6. CD45 T and CD20 B cells formed inflammatory clusters close to the edge of and around vessels of the EAT sections in the simvastatin group, but the center of the fat fragment was free of inflammatory cells. 200X. See text for details. **(B)** Immunohistochemical staining for pro- and anti-inflammatory cytokines in the EAT. CD68+: macrophages; CD45 T: T-lymphocytes; CD20 B: B-lymphocytes; TNF-α: tumoral necrosis factor alpha; IL-6: interleukin 6. CD45 T and CD20 B cells formed inflammatory clusters close to the edge of and around vessels of the EAT sections in the simvastatin group, but the center of the fat fragment was free of inflammatory cells. 200X. See text for details.

Pioglitazone alone and simvastatin + pioglitazone treatment were associated with a lower mean percentage positive area of CD68+/macrophages, CD45/T-lymphocytes, TNF-α, IL-6, leptin and resistin in the EAT fragments compared to controls (p < 0.001). Conversely, a significantly higher mean percentage positive area for adiponectin was observed (Figures 1 and 2). No significant differences in the mean percentage positive area for CD20/B-lymphocytes were observed between pioglitazone alone and the control group (Figure 1).

Some morphological aspects are noteworthy. Inflammatory cells and cytokines were distributed over the adipose tissue area in the control group. CD45 T-lymphocytes and CD20 B-lymphocytes formed inflammatory clusters close to the edge and around vessels of the adipose tissue in the simvastatin group, but the center of the fat fragment was free of inflammatory cells (Figure 2). Therefore, the mean percentage positive area was larger in the simvastatin group compared to the control group (p < 0.05) (Figure 1).

### Correlation between plasma values and tissue biomarkers

**Figure 3:**
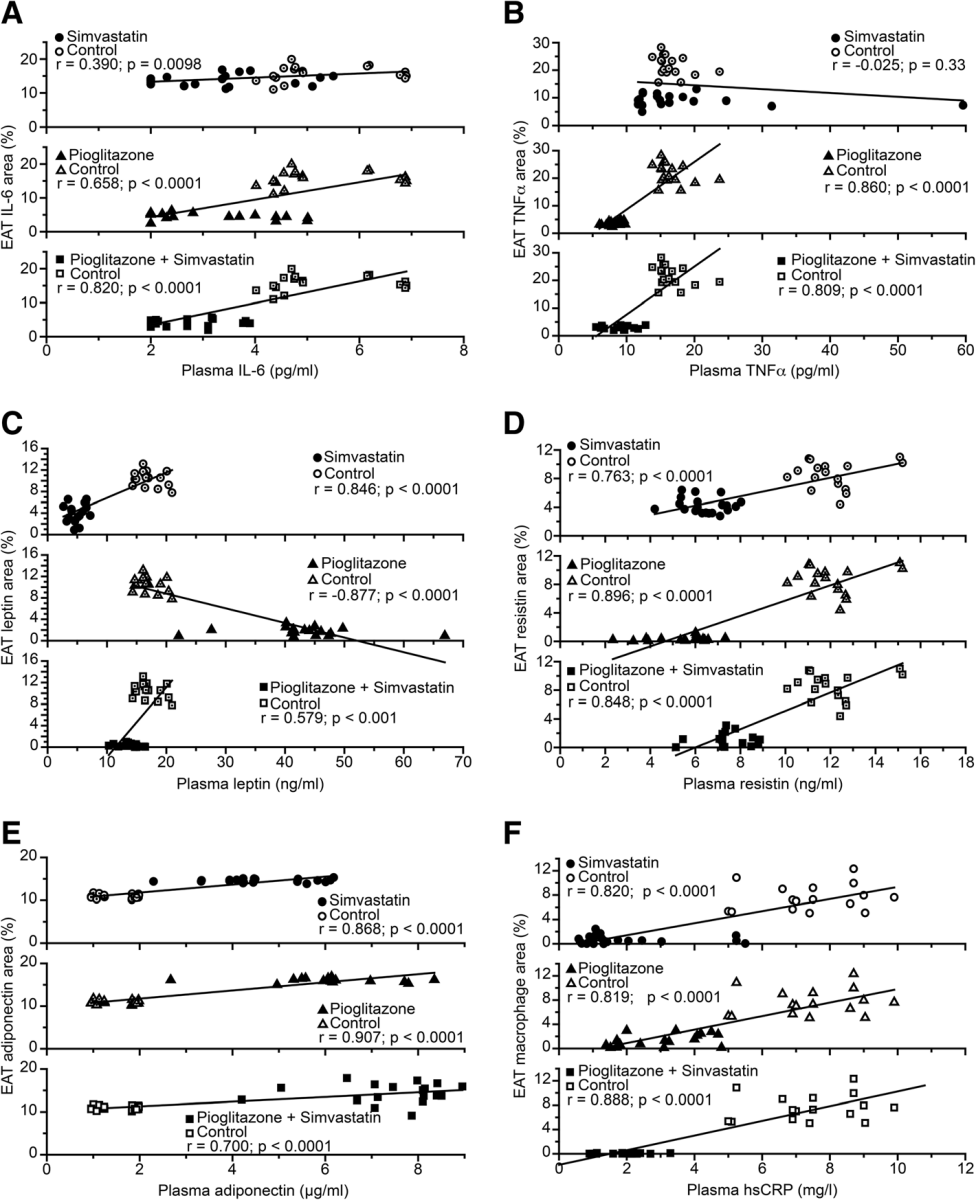
**Correlations between plasma and EAT inflammatory variables. (A)** IL-6; **(B)** TNF-α; **(C)** Leptin. **(D)** Resistin; **(E)** Adiponectin **(F)** EAT macrophages and plasma hsCRP. TNF-α: tumor necrosis factor alpha; IL-6: interleukin 6; hsCRP: high-sensitivity C-reactive protein. See text for details.

We investigated whether the treatment effects on plasma biomarkers of inflammation produced corresponding effects on EAT. Positive correlations between plasma cytokines and correspondent cytokines in EAT were found after simvastatin, pioglitazone and pioglitazone + simvastatin treatments (Figure 3). TNF-α after treatment with simvastatin (r = -0.025, p = 0.33) and leptin after treatment with pioglitazone (r = -0.877, p < 0.0001) showed negative correlations. A positive correlation was found between serum hsCRP and the percentage of macrophages in EAT, which indicates simultaneous reductions in plasma and tissue values (Figure 3F). No correlations between plasma lipid variables and tissue inflammatory variables were observed.

## Discussion

This study found that macrophages infiltration and pro-inflammatory cytokines, TNF-α, IL-6, leptin and resistin, were decreased in the EAT of CAD/MS patients treated with simvastatin or pioglitazone, either as monotherapy or in combination. Furthermore, these treatments were also associated with an increased presence of adiponectin, an anti-inflammatory cytokine secreted by EAT. These findings reflected the observations in plasma because a positive correlation between the percentage area of macrophages in EAT and plasma hsCRP after treatments was observed. T- and B-lymphocytes and macrophage clusters were concentrated near the edge or around blood vessels in EAT fat fragments of patients treated with simvastatin, which is a novel observation.

Simvastatin + pioglitazone treatment was associated with significantly lower plasma hsPCR, IL-6, TNF-α, resistin, ADMA and MMP-9 compared to the control group. Higher plasma adiponectin and lower hsCRP concentrations in this group occurred simultaneously, which indicated a predominance of the anti-inflammatory effect. The lowest plasma leptin, resistin and MCP-1 values were found in patients treated with simvastatin alone. These findings are consistent with Jialal et al. [21], who observed that pravastatin, atorvastatin or simvastatin therapy significantly reduced hsCRP levels, which further supports an anti-inflammatory effect of statins. The PIOSTAT Study [22] found that, non-diabetic patients with cardiovascular disease and elevated hsCRP levels showed significant anti-inflammatory effects of pioglitazone that were comparable to 40 mg simvastatin. Furthermore, these authors showed that pioglitazone + simvastatin had additive effects on hsCRP with a reduction greater than 40%, but only for patients without MS. Pioglitazone, as a monotherapy and in combination, significantly reduced MMP-9 [23]. MMP-9 levels were also elevated in patients with diabetes mellitus, and treatment with glitazones effectively reduced the levels of this enzyme [24,25].

We noted that therapeutic interventions that interfere with plasma cytokines and inflammatory markers also modify the inflammatory response in EAT of CAD/MS patients. Therefore, the mean percentage positive area with macrophages, T-lymphocytes, TNF-α, IL-6, leptin and resistin was significantly lower in EAT fragments after 90 days of pioglitazone only or simvastatin + pioglitazone therapies than the control group. EAT is a source of inflammatory cytokines, and this finding is considerably important. An augmented inflammatory response is associated with significant macrophage infiltration, which makes local adipose tissue deposits a cardiovascular risk factor [6,7,9]. This hypothesis is supported by Suganami and Hirata [9,26], who suggested that a vicious cycle of infiltrating macrophages and adipocytes augments the inflammatory response in adipose tissue, especially in obesity-associated metabolic abnormalities, such as insulin resistance and diabetes. CAD patients with MS and type 2 diabetes were treated with pioglitazone, and this therapy was associated with decreased IL-1β, IL-1Ra and IL-10 mRNA expression in EAT. Pro- and anti-inflammatory genes were differentially increased in EAT and selectively reduced following pioglitazone treatment [27].

We observed that the positive area for adiponectin was five times lower in CAD/MS untreated patients than after pioglitazone alone or simvastatin + pioglitazone therapies. Furthermore, positive correlations were found between plasma cytokines and correspondent cytokines in EAT after simvastatin, pioglitazone and pioglitazone + simvastatin treatments. A positive correlation between plasma hsCRP and the mean percentage of area to macrophage in EAT was also shown. TNF-α after simvastatin treatment and leptin after pioglitazone treatment were negatively correlated, which indicated a differential effect in tissue and plasma. The decreased leptin in EAT following TZD treatment demonstrates adipose depot-specific responsiveness [28,29] or alternatively indicates that TZDs induce translational or posttranslational changes that increase protein levels without increasing mRNA levels [30]. The high leptin plasma concentrations in these circumstances are most likely due to production from subcutaneous adipose tissue [28]. However, Iacobelis et al. showed significantly lower adiponectin expression in epicardial fat isolated from patients with CAD [31]. Ouchi et al. observed a significant inverse correlation between CRP and adiponectin mRNA levels in human adipose tissue from patients with documented coronary atherosclerosis [32]. Patients with MS expressed lower EAT adiponectin levels than patients without MS [33]. Iacobellis et al. showed peripheral adiponectin levels and epicardial fat adiponectin protein expression were the best correlates of left coronary artery adiponectin,. They showed that intracoronary adiponectin levels reflect systemic adiponectin levels. Epicardial adipose tissue could partially contribute to adiponectin levels in the coronary circulation [34], although that intracoronary plasma adiponectin rapidly and significantly increases in patients with CAD after CABG [35].

We also showed that T- and B-lymphocytes and macrophage clusters concentrated near the edge or around blood vessels in fat fragments of patients treated with simvastatin alone, but the center of the fat fragments was free of inflammatory cells. One possible explanation for these findings is that cell residues were driven to tertiary lymphoid organs, which are ectopic accumulations of lymphoid cells that arise under environmental influences, especially during chronic inflammation. This hypothesis is supported by the observation that prolonged inflammatory cytokine production and/or lymphoid chemokine expression is sufficient to induce lymphoid neogenesis [36]. Additionally, lymph nodes during inflammation are characterized by an increase in blood flow and T- and B-lymphocyte migration [37]. Finally, clinical therapies can reverse the clusters of lymphoid cells via cleansing of the inflammation-inducing agent [38].

The present study demonstrated the novel ability of simvastatin and pioglitazone to reduce plasma and tissue inflammation simultaneously. This finding may represent one mechanism that these drugs protect the cardiovascular system against hypercholesterolemia and hyperglycemia.

These observations extend our understanding of the actions of therapeutic interventions that might interfere with EAT and potentially contribute to the control mechanisms involved in the pathogenesis of coronary atherosclerosis and decrease the cardiovascular risk.

### Study limitations

The sample size was small, but it was sufficient for the proposed objective. The results do not allow us to establish firm conclusions about drug efficacy because this was a non-randomized trial without pre- and post-treatment data. However, this analysis was not the main objective of the study. Rather, we were primarily interested in the correlations of plasma/tissue actions. We found significant positive correlations between plasma and EAT effects of simvastatin, pioglitazone and their combination.

## Conclusions

Pioglitazone, simvastatin or combination treatment in CAD and MS patients substantially reduced epicardial adipose tissue and plasma inflammatory markers. These tissue effects may contribute to the control of coronary atherosclerosis progression and may be inferred from plasmatic findings.

## Competing interests

The authors declare that they have no competing interests.

## Authors’ contributions

AFG – performed essentially all the experiments, data analysis and writer of the study. SFO – contributed to the design and to study planning. DF – contributed to design of the study and performed statistical analysis. MLH – intelectual contribution to study planning; immunohistochemical staining analysis were performed in her laboratory. LAOD – contributed to the obtain adipose tissue samples during surgery elective bypass grafting. PLL – Head of the research group, mentor and writer of the study. All authors read and approved the final manuscript.
